# Mitochondrially-targeted expression of a cytoplasmic male sterility-associated *orf220 *gene causes male sterility in *Brassica juncea*

**DOI:** 10.1186/1471-2229-10-231

**Published:** 2010-10-26

**Authors:** Jinghua Yang, Xunyan Liu, Xiaodong Yang, Mingfang Zhang

**Affiliations:** 1 Laboratory of Genetic Resources & Functional Improvement for Horticultural Plants, Department of Horticulture, Zhejiang University, Hangzhou, 310029, P. R. China; 2 Laboratory of Horticultural Plant Growth, Development & Quality Improvement, Ministry of Agriculture, Hangzhou, 310029, P. R. China

## Abstract

**Background:**

The novel chimeric open reading frame (*orf*) resulting from the rearrangement of a mitochondrial genome is generally thought to be a causal factor in the occurrence of cytoplasmic male sterility (CMS). Both positive and negative correlations have been found between CMS-associated *orfs *and the occurrence of CMS when CMS-associated *orfs *were expressed and targeted at mitochondria. Some *orfs *cause male sterility or semi-sterility, while some do not. Little is currently known about how mitochondrial factor regulates the expression of the nuclear genes involved in male sterility. The purpose of this study was to investigate the biological function of a candidate CMS-associated *orf220 *gene, newly isolated from cytoplasmic male-sterile stem mustard, and show how mitochondrial retrograde regulated nuclear gene expression is related to male sterility.

**Results:**

It was shown that the ORF220 protein can be guided to the mitochondria using the mitochondrial-targeting sequence of the *β *subunit of F1-ATPase (*atp2-1*). Transgenic stem mustard plants expressed the chimeric gene containing the *orf220 *gene and a mitochondrial-targeting sequence of the *β *subunit of F1-ATPase (*atp2-1*). Transgenic plants were male-sterile, most being unable to produce pollen while some could only produce non-vigorous pollen. The transgenic stem mustard plants also showed aberrant floral development identical to that observed in the CMS stem mustard phenotype. Results obtained from oligooarray analysis showed that some genes related to mitochondrial energy metabolism were down-regulated, indicating a weakening of mitochondrial function in transgenic stem mustard. Some genes related to pollen development were shown to be down-regulated in transgenic stem mustard and the expression of some transcription factor genes was also altered.

**Conclusion:**

The work presented furthers our understanding of how the mitochondrially-targeted expression of CMS-associated *orf220 *gene causes male sterility through retrograde regulation of nuclear gene expression in *Brassica juncea*.

## Background

Cytoplasmic male sterility (CMS), the maternally inherited trait of failure to produce functional pollen, exists in many plant species and has wide application for the production of hybrid crops. CMS can occur at different stages during reproductive development. It is generally believed that CMS is associated with the rearrangement of mitochondrial genomes, which, in many cases, is attributed to the generation of novel open reading frames (*orf*s) [1-5]. Some experimental evidence confirms the correlation between CMS-associated *orfs *and the occurrence of CMS. In some studies mitochondrially-targeted expression of novel *orfs *was shown to lead to male sterility or semi-sterility [6-9], while in others it did not [10-12]. A probable interaction between *orfB *and the ATP synthesis complex in CMS has been demonstrated in sunflower using 2-D electrophoresis and Western blot analysis [13]. However, the specific role of mitochondrial novel *orfs *in causing male sterility is not yet clearly established and better evidence that mitochondrially-targeted expression of *orfs *causes male sterility is needed. In particular, how mitochondrial factor regulates the expression of the nuclear genes involved in male sterility is poorly understood. Is there any cross-talk between mitochondria and the nucleus that ultimately determines the abortion of pollen? If so, how do mitochondrial factors directly or indirectly halt the processes of pollen development, and through which pathway?

Recently, many studies have focused on mitochondrial regulation of nuclear gene expression in higher plants [14-18]. This communication pathway from mitochondria to the nucleus is defined as mitochondrial retrograde regulation (MRR), and has been documented mainly in yeast and animals [19,20]. Some ABC model genes related to floral organ development, namely the nuclear MADS-box TF genes, have been shown to be targets for floral organ homeotic transformation regulated by MRR [21-26]. In addition, several other nuclear genes have recently been shown to be retrograde regulated by mitochondria in some CMS systems [27-29].

Previously, we isolated the CMS-associated *orf220 *gene from CMS stem mustard, *Brassica juncea*, [30]. In the present study, we constructed transgenic stem mustard expressing the chimeric *orf220 *gene mediated by *Agrobacterium tumefaciens*. These transgenic stem mustard plants exhibited male sterility. Global changes in the expression of mitochondrial and nuclear genes in transgenic stem mustard were examined using oligoarray analysis.

## Results

### Chimeric gene construction and transformation of stem mustard

**Figure 1:**
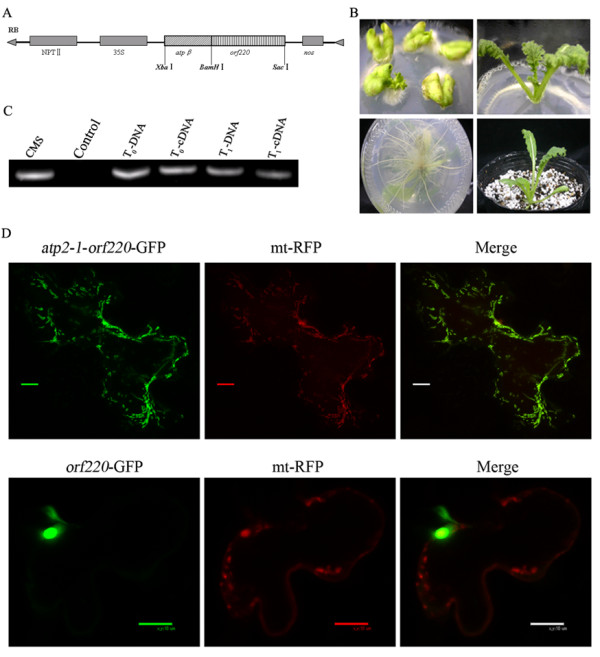
**Construction of chimeric *orf220 *gene, transformation into wild type stem mustard and its sub-localization in mitochondria**. *Orf220*, a candidate cytoplasmic male gene associated with sterility in *Brassica juncea*, was isolated from cytoplasmic male-sterile stem mustard. A mitochondrial targeted peptide (*atp2-1*) was isolated from *Nicotiana *encoding the *β *subunit of F1-ATPase (Figure 1-A). Figure 1-B shows the transgenic regenerations from proximal portions of hypocotyls of cotyledons in stem mustard. Figure 1-C shows the identification of transgenic stem mustard by checking for the presence of the *orf220 *gene using PCR and RT-PCR. Figure 1-D shows that the mitochondrial targeted peptide (*atp2-1*) can guide the ORF220 protein to the mitochondria target, while the ORF220 protein is only found in the nucleus in the absence of the mitochondrial targeted peptide (*atp2-1*). Mt-RFP was used as the mitochondrially-targeted control.

We had previously isolated the CMS-associated gene, *orf220*, from CMS stem mustard (Zhang et al., 2003). To verify the function of this gene, a chimeric gene was constructed by fusing *orf220 *with a mitochondrial-targeting sequence derived from *atp2-1 *and amplified from tobacco (*Nicotiana plumbaginifolia*). The *orf220 *gene was amplified from CMS stem mustard and ligated after the mitochondrial-targeting sequence (*atp2-1*). The chimeric gene was then ligated to the transferred DNA binary vector, pBI121, driven by the 35 S promoter. The schematic structure of the chimeric *orf220 *gene is shown in Figure 1-A. The constructs were then introduced into *A. tumefaciens *LBA4404.

The transformation system for stem mustard established in our laboratory is based on a regeneration system from cotyledons with proximal hypocotyls (Figure 1-B). We used this transformation system to produce transgenic stem mustard plants incorporating the alien chimeric *orf220 *gene. Candidate transgenic stem mustard plants were checked by PCR and RT-PCR (Figure 1-C) and 5 T_0 _plants were selected, all exhibiting male sterility. We selected one of these transgenic lines and obtained 28 T_1 _generation plants, which yielded 13 male-sterile and 15 male-fertile plants. We also used the *orf220 *gene as a marker to check that this gene was inherited and could be expressed in T_1 _transgenic stem mustard. The male-sterile phenotype was shown to be genetically transmitted to the T_1 _generation and associated with *orf220 *expression (Figure 1-A). All the 13 male-sterile T_1 _plants expressed the *orf220 *gene, and all the 15 male-fertile T_1 _plants lacked *orf220 *expression (data not shown).

We used the chimeric *orf220 *gene with GFP expression to check whether this chimeric gene was targeted to the mitochondria. It was shown that the *orf220 *gene with the mitochondrial-targeting sequence (*atp2-1*) was located in the mitochondria, however, the *orf220 *gene without the mitochondrial-targeting sequence (*atp2-1*) was only found in the nucleus (Figure 1-D).

### Phenotypes of transgenic stem mustard

**Figure 2:**
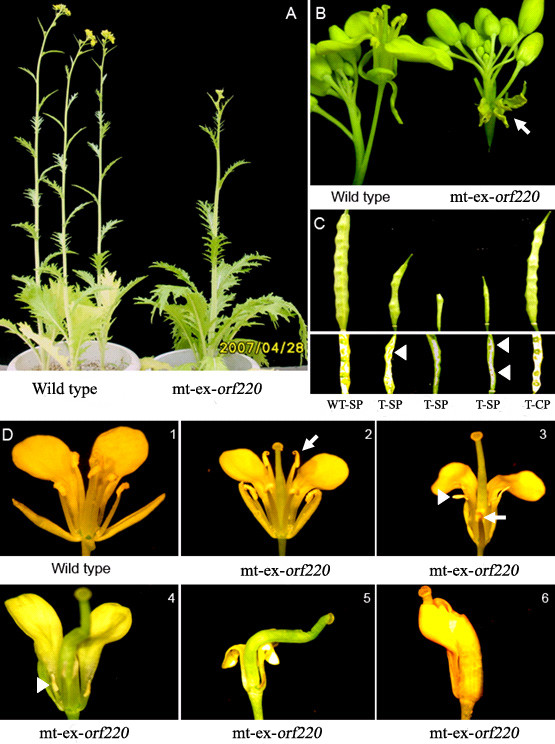
**Phenotypes of reproductive development in transgenic plants**. Delayed flowering of transgenic plants is shown in Figure 2-A. The appearance of novel petal-like floral structures in transgenic plants is shown by an arrow with a hand in Figure 2-B. The time of seed-set of transgenic plants is shown by an arrow without a hand in Figure 2-C. Transgenic plant stamens with pollen are shown by an arrow with a hand, and stamens without by an arrow without a hand in Figure 2-D. WT denotes wild type; mt-ex-*orf220 *denotes mitochondrial targeted expression of *orf220 *gene; SP denotes self-pollination; CP denotes cross-pollination with normal pollen.

Alterations to the phenotype were observed in transgenic plants in which the chimeric *orf220 *gene was expressed. The phenotype was stably inherited between generations. The vegetative growth of transgenic stem mustard was similar to that of WT plants, but their reproductive characteristics differed in numerous ways. Transgenic plants exhibited: 1) 2-3 day delays in bolting and flowering time (Figure 2-A); 2) the appearance of novel, petal-like floral structures at the basal part of the buds (Figure 2-B); and 3) a lack of seed-set suggesting sterility, even though an episperm was observed at an early developmental stage in some siliques (Figure 2-C). When transgenic plants were cross-pollinated with WT pollen, seed-setting occurred indicating normal pistil fertility, and the retention of normal pistil function in transgenic plants (Figure 2-C). Five types of aberrant flowers were observed in transgenic stem mustard: 1) flowers with six normal stamens (Figure 2-D2); 2) flowers having two stamens with pollen, and four without pollen (Figure 2-D3); 4) flowers with degenerative stamens devoid of pollen and with crooked pistils (Figure 2-D4, 5); and 5) flowers with a closed corolla and a pistil on the outside (Figure 2-D6). Each transgenic plant bore all five types of aberrant flower. Importantly, the phenotypes of aberrant floral organs in transgenic plants were shown to be identical with those of CMS stem mustard (supplementary data additional file 1 and [31]). The expression of *orf220 *without mitochondrial-targeting peptides did not result in a CMS phenotype in *Arabidopsis *(data not shown).

### Activity of pollen produced from transgenic stem mustard

**Figure 3:**
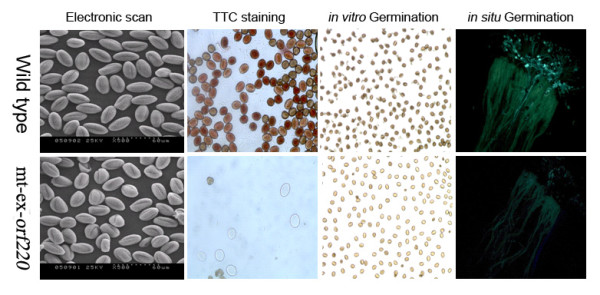
**Morphology and activity of pollen produced from transgenic and wild type plants**. mt-ex-*orf220 *denotes the mitochondrial targeted expression of *orf220 *gene. TTC denotes 2,3,5-triphenyl-2h-tetrazolium chloride.

However, some stamens from transgenic plants can still produce pollen and morphological screening, *in vitro *germination and *in-situ *pollen germination were performed to determine the activity of this pollen. The morphology of pollen from transgenic plants was altered (Figure 3). While most of the pollen produced by WT plants was susceptible to staining with TTC, some transgenic stem mustard pollen grains resisted staining with TTC, indicating that pollen activity of transgenic plants was markedly different (Figure 3). After 4 hrs of *in vitro *germination, pollen from WT plants was able to produce pollen tubes. However, pollen from transgenic stem mustard failed to germinate (Figure 3). After 12 hrs of *in-situ *germination on the stigma, fluorescence signals could be detected in pollen from WT plants, but this was not the case for pollen from transgenic stem mustard (Figure 3).

### Global gene expression patterns in transgenic and WT stem mustard

**Figure 4:**
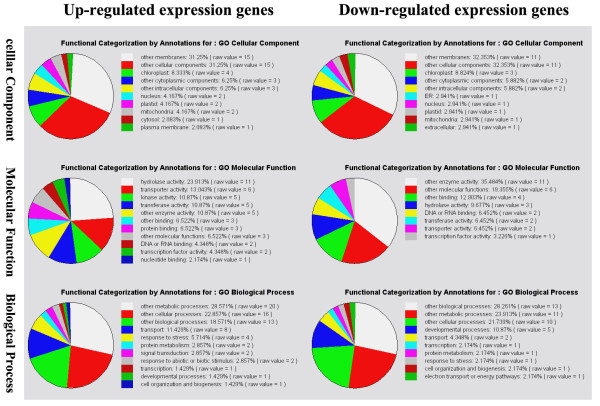
**Classification of genes expressed according to their cellular components, molecular function and biological processes in transgenic plants**. Up-regulated and down-regulated genes expressed in transgenic stem mustard plants.

To discover which gene clusters in the mitochondria and nucleus are responsive to mitochondrially-targeted expression of the *orf220 *gene, we compared global gene expression patterns between WT and transgenic stem mustard using an oligoarray analysis. Transgenic stem mustard genes that were either down-regulated or up-regulated more than two-fold, were selected and sorted according to their cellular components, molecular function and biological processes (Figure 4).

#### Down-regulated Genes

**Figure 5:**
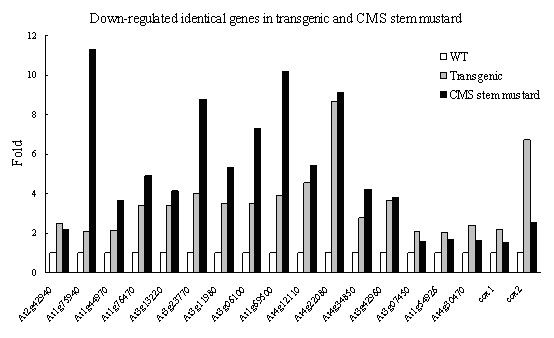
**Identical down-regulated genes in transgenic and CMS stem mustard**. WT indicates wild type.

Some examples of genes that were down-regulated in transgenic stem mustard are shown in Table 1. A number of mitochondrial genes, such as *cox1*, *cox2 *and *atp9*, were down-regulated to different degrees in transgenic stem mustard. At2g22080, a gene for a pectate lyase family protein, which is an enzyme involved in the maceration and soft rotting of plant tissue via degradation of the cell wall, was down-regulated more than 8-fold in transgenic stem mustard. At1g01280 and At1g69500 encode for cytochrome P450 family proteins which function as mono-oxygenases and are involved in hormone, phenolic, alkaloid, sterol and oxygenated fatty acid synthesis. At2g42840 encodes protodermal factor1 (*PDF1*) protein involved in early meiosis and shoot meristem development. At1g75940 and At3g23770 are male fertility related genes coding for proteins in the glycosyl hydrolase family, with anther-specific expression. At3g11980 is a male-sterile gene, which encodes for male sterility protein 2 (*MS2*), and for a transcriptional factor gene reported to affect pollen development in *Arabidopsis*. At3g06100, is a gene for a MIP family protein, and is preferentially expressed in the surrounding sporophytic tissues of stamens. At4g12110 encodes a sterol desaturase family protein, which acts as a mediator of fertility. Genes for an ABC transporter family protein (At3g13220), a calcium-binding EF hand family protein (At3g18430) and an alcohol dehydrogenase (At3g42960) were also down-regulated in transgenic stem mustard, as were some genes (At2g42940, At1g76470, At3g07450, At1g20370, At4g30470 and At4g34850) that have been reported to be associated with pollen development. All of the down-regulated genes detected in transgenic plants are listed in supplementary data (additional file 2). The expression of 168 genes was down-regulated in CMS mustard, while 30 genes were down-regulated in transgenic plants, only 17 of these genes being in common (Figure 5).

**Table 1:** Down-regulated expressed genes in transgenic stem mustard observed in this study

Gene ID	Gene Description	Fold
At2g42940	DNA-binding family protein	2.5
At2g42840	protodermal factor 1 (PDF1)	4.6
At1g75940	glycosyl hydrolase family 1 protein/anther-specific protein ATA27	2.09
At1g01280	cytochrome P450 family protein	3.59
At1g76470	cinnamoyl-CoA reductase family	3.41
At3g07450	protease inhibitor/seed storage/lipid transfer protein (LTP) family protein	2.1
At3g18430	calcium-binding EF hand family protein	2.03
At3g13220	ABC transporter family protein	3.39
At3g23770	glycosyl hydrolase family 17 protein	3.98
At3g11980	male sterility protein 2 (MS2)	3.48
At3g06100	major intrinsic family protein/MIP family protein	3.51
At1g69500	cytochrome P450 family protein	3.89
At1g20370	tRNA pseudouridine synthase family protein	2.08
At4g12110	sterol desaturase family protein	4.56
At4g22080	pectate lyase family protein/pectate lyase family protein	8.66
At4g30470	cinnamoyl-CoA reductase-related	2.42
At4g34850	chalcone and stilbene synthase family protein	2.79
At3g42960	alcohol dehydrogenase (ATA1)	3.62
cox2	cytochrome c oxidase subunit 2	6.72
cox1	cytochrome c oxidase subunit 1	2.19
mitochondria.1	ATP synthase subunit 9	2.06

#### Up-regulated Genes

Representative examples of genes that were up-regulated in transgenic stem mustard are shown in Table 2. At2g02930 and At4g25100 encode for glutathione *S*-transferase and superoxide dismutase, respectively, and maintain homeostasis in plant cells. At3g14450 and At3g19090 are thought to code for RNA binding proteins. Some kinase genes were up-regulated (At2g20900, At4g39940, At5g12000 and At4g15530). At3g61890 is a gene for homeobox-leucine zipper protein 12. At5g25560 encodes a zinc finger family protein. Some genes related to metabolism were also up-regulated (At1g78000, At1g32780, At1g14420, At1g12240 and At5g20710), as well as At1g52940, a gene for a calcineurin-like phosphoesterase family protein. All of the up-regulated genes detected in transgenic stem mustard are listed in supplementary data (additional file 3).

**Table 2:** Up-regulated expressed genes in transgenic stem mustard observed in this study

Gene ID	Gene Description	Fold
At2g02930	glutathione S-transferase, putative/glutathione S-transferase, putative	3.03
At2g20900	diacylglycerol kinase, putative	2.14
At1g78000	sulfate transporter (Sultr1;2)	2.11
At1g32780	alcohol dehydrogenase, putative	2
At1g14420	pectate lyase family protein	2.12
At1g52940	calcineurin-like phosphoesterase family protein	2.11
At1g12240	beta-fructosidase (BFRUCT4)/beta-fructofuranosidase/invertase, vacuolar	2.04
At3g14450	RNA-binding protein, putative	2.28
At3g19090	RNA-binding protein, putative	2.21
At4g25100	superoxide dismutase (Fe), chloroplast (SODB)/iron superoxide dismutase (FSD1)	2.57
At4g39940	adenylylsulfate kinase 2 (AKN2)	2.06
At3g61890	homeobox-leucine zipper protein 12 (HB-12)/HD-ZIP transcription factor 12	2.13
At5g12000	protein kinase family protein	2.48
At5g25560	zinc finger (C3HC4-type RING finger) family protein	2.31
At5g20710	beta-galactosidase, putative/lactase, putative	2.11
At4g15530	pyruvate phosphate dikinase family protein	2.67

## Discussion

The precise mechanism by which mitochondria trigger male sterility is still unknown. It is well known that there is a relationship between novel *orf*s and the occurrence of CMS, in which *orf*s play an essential role in disrupting mitochondrial function [1-5]. Meanwhile, several recent studies using cDNA microarrays have identified some nuclear target genes downstream of the pathway of CMS occurrence [27,28]. However, the exact mechanism underlying the occurrence of CMS, especially the downstream nuclear target genes and retrograde signaling pathway, remains to be exploited.

The evidence varies as to whether mitochondrially-targeted expression of novel *orf*s can induce male sterility or not. In some cases this leads to male sterility and in others to semi-sterility [6-9]. However, some mitochondrially-targeted expressions of such novel *orf*s fail to induce either male sterility or semi-sterility [10-12]. The failure of mitochondria-target expression of novel *orf*s to induce male sterility is probably due to problems of sub-mitochondrial location [12], the expression period [11] or the expression amount of ORF protein [10] in transgenic plants. In the present study, the reproductive phenotype of transgenic stem mustard was extremely similar to those observed in CMS stem mustard ([31] and supplementary data, Figure 1). From these observations, we concluded that ectopic expression of the chimeric *orf220 *gene causes male sterility in transgenic stem mustard. And this could be attributed to direct mitochondrial localization of ORF220 protein guided by a mitochondrial-targeting peptide. In transgenic plants, we observed reduced expressions of several mitochondrial genes related to respiratory complex, as caused by ectopic expression of chimeric *orf220 *gene, which may affect mitochondrial function, although we don't know how this happens.

Functional genes specifically related to pollen development have been well documented till now, of which mutation of any of these genes causes failure of microsporogenesis or abortion of pollen [32-34]. In our study, we observed that many genes related to pollen development were down-regulated in transgenic plants. Furthermore, many same genes in relation to mitochondrial respiratory complex and pollen development were also observed to be down-regulated in transgenic plants and CMS line as well, although we couldn't conclude that these identical down-regulated genes were the causal factor of producing similar phenotype in transgenic plant and CMS line from this study. It is unlikely that mitochondrial proteins alone could directly result in male sterility without a signal transduction from the mitochondria to the nucleus. Such signaling from the mitochondria to the nucleus, termed mitochondrial retrograde regulation, has been well described in yeast and mammals [19,20], although it is less frequently reported in higher plants. Among the clusters of expressed genes in transgenic and WT plants, we also observed several transcriptional factor (TF) genes were induced by ectopic expression of chimeric *orf220 *gene, such as, homeobox-leucine zipper family protein (HD-ZIP), DNA/RNA binding protein and zinc finger family protein etc. These TF genes were reported to be involved in the regulation of developmental processes, the response of plants to environmental and redox regulation [35-39]. However, whether these TF genes are associated with mitochondrial retrograde regulation of nuclear gene expression or not need to be further substantiated.

## Conclusion

In conclusion, the mitochondrially-targeted expression of *orf220 *gene was capable of inducing male sterility in transgenic stem mustard. We proposed that the transformation of *orf220 *gene in stem mustard impairs mitochondrial function, and this response is signaled by the mitochondria to nucleus through a particular signal transduction pathway. How the *orf220 *gene functions precisely to induce male-sterility and its biochemical characterization remains to be discovered. In addition, further research is worthy of being investigate to explore the signal pathway of mitochondrial retrograde regulation and how nuclear target genes are responsive in order, expecially what is the primary receptor gene in the nucleus, if any.

## Methods

### Construction and transformation of the chimeric orf220

The candidate CMS-associated *orf220 *gene was cloned from CMS stem mustard using a pair of primers in which a *BamH*I site was introduced at the 5' end, and a *Sac*I site at the 3' end. The mitochondrial-targeting sequence (from *atp2-1*) was amplified from tobacco using primers that introduced an *Xba*I site at the 5' end and a *BamH*I site at the 3' end. The mitochondrial transit sequence was the N-terminal 60 amino acid sequence of *atp2-1*, a peptide which targets proteins foreign to the mitochondria and is cleaved between the 54^th ^and 55^th ^amino acid sites after translocation in transgenic plants [6,10]. The procedures used to construct the chimeric gene followed He *et al*. [6]. This chimeric T-DNA was then transformed into *A. tumefaciens *LBA4404 as described below. A schematic diagram of the chimeric gene vector is shown in Figure 1-A, and the primers used are listed in Table 3.

**Table 3:** Primers used in chimeric expression vector construction

Genes	F/R	Primers Sequence (5'-3')
*orf220*	F	ATGCCTCAACTGGATAAATTCACTT
	R	TCATCGAAATAGATCGAGGATCTCG
*Chimeric gene orf220*	F	TAC*GGATCC*ATGCCTCAACTGGAT (*BamH*I)
	R	AGA*GAGCTC*TCATCGAAATAGATC (*Sac*I)
*Chimeric gene atp2-1*	F	CC*TCTAGA*CCATGGCTTCTCGGAGGCTTCT (*Xba*I)
	R	CC*GGATCC*GCTGCGGAGGTAGCGTACTG (*BamH*I)
*atp2-1*/*orf220 *GFP	F	CACCATGGCTTCTCGGAGGCTTCT
	R	TCGAAATAGATCGAGGATCTCG
*orf220 *GFP	F	CACCATGCCTCAACTGGATAAATTCACTT
	R	TCGAAATAGATCGAGGATCTCG

Note: underlined italicized nucleotides represent restriction enzyme sites. F, forward; R, reverse.

Proximal portions of hypocotyls from cotyledons obtained from 5-day-old aseptic seedlings were pre-cultured (MS + 3 mg/L 6-BA + 0.5 NAA + 3% sucrose + 0.8% agar) for 2 days, and subsequently co-cultured with *A. tumefaciens *containing the chimeric gene in the dark for 2 days on differentiation medium (MS + 3 mg/L 6-BA + 0.5 NAA + 3% sucrose + 0.8% agar). Shoots were subsequently regenerated for resistance screening from the proximal portions of the cotyledon hypocotyls on differentiation media supplemented with kanamycin (MS + 3 mg/L 6-BA + 0.5 NAA + 20 mg/L kanamycin + 3% sucrose + 0.8% agar). Regenerating shoots (5 cm in length) were cut from explants and rooted in the following medium: 1/2 MS + 0.1 NAA + 10 mg/L kanamycin + 3% sucrose + 0.8% agar. This regeneration system from stem mustard cotyledons was developed in our laboratory [40].

### Construction of GFP fusion vectors and transient expression

The chimeric *atp2-1*/*orf220 *and *orf220 *coding sequences were amplified from the chimeric vector obtained above using standard protocols with the LA Taq PCR system (Takara, Japan), and using specific primers flanked by Gateway recombination cassettes (Invitrogen, California, USA). The primers used here are listed in Table 3. PCR products were cloned into pDONR221 according to the manufacturer's instructions. Cloning into the final GFP vectors (pK7FWG2) was by LR reaction (Invitrogen, California, USA). The mt-RFP plasmid containing the pre-sequence of *Arabidopsis thaliana *ATPase delta-prime subunit and DsRed2 was provided by Dr. S. Arimura and Prof N. Tsutsumi (Laboratory of Plant Molecular Genetics, The University of Tokyo) [41].

Biolistic co-transformation of the GFP and RFP fusion vectors was performed on *Arabidopsis *leaves. In brief, GFP and RFP plasmids (5 μg each) were co-precipitated onto gold particles and transformed using a PDS-100/He biolistic transformation system (Bio-Rad, http://www.bio-rad.com). Healthy *Arabidopsis *leaves were placed on MS medium and bombarded. Leaves were then incubated for 48 hrs at 22°C before microscopy using a Nikon fluorescence microscope system.

### Phenotypic evaluation of transgenic stem mustard

Regenerated maintainer lines of stem mustard plants from medium containing kanamycin (50 mg/L) were treated as putative candidates and were further screened by rooting them on medium containing kanamycin (25 mg/L). Putative transgenic stem mustard, designated as T_0_, with normal roots was identified using the *orf220 *gene in PCR and RT-PCR analysis. At flowering, they were pollinated with normal pollen from WT plants. Seeds from the T_0 _generation were grown as T_1 _generation and were further identified using the *orf220 *gene in PCR and RT-PCR analysis. The primers used in these analyses are listed in Table 3. Transgenic stem mustard plants were observed at flowering time. Seeds were set to study male fertility by self-pollination using a bag covering the flower. Female fertility of transgenic plants was confirmed by pollination with WT pollen.

### Pollen morphology and activity evaluation

The morphology of pollen grains of transgenic and WT stem mustard was examined using a scanning electron microscope (KYKY-1000B). Pollen activity was evaluated using 2,3,5-triphenyl-2h-tetrazolium chloride (TTC) staining, *in vitro *germination and *in-situ *germination. In TTC staining, pollen grains were soaked in 0.1% TTC solution. Active pollen stains red because the NADH/NADPH produced deoxidizes TTC to TTF (which is red). Pollen *in vitro *germination was performed at 28°C and 100% relative humidity, during which pollen grains were cultured on a liquid medium consisting of boric acid (250 mg/L) and sucrose (10%). Pollen germination success was calculated and photographed after 4 hrs using a microscope (LEICA). To assess *in-situ *germination, pollen from transgenic and WT plants were placed onto the surface of the stigma and 12 hrs after pollination the pistils were removed and fixed rapidly in FAA fixing solution (ethanol: acetic acid, 3:1) for 2 hrs. The fixed pistils were washed three times with sterile water and treated overnight in softening solution (8 mol/L NaOH). The pistils were then washed in distilled water and stained in 0.1% aniline blue for 3 hrs in the dark. The stained pistils were observed and photographed with a Leica DMRA2 fluorescent microscope.

### Oligoarray analysis

Floral buds of one inflorescence from WT, transgenic stem mustard, and CMS stem mustard were collected to compare the expression of genes during floral development. Bud samples were ground in liquid nitrogen, and total RNAs were prepared using Trizol reagent according to the manufacturer's protocol (Invitrogen). The oligoarrays used in this study were derived from the *Arabidopsis thaliana *ATH1 chip. All the hybridization procedures and data analysis were performed by CapitalBio Corp. (Bejing, China). Arrays were scanned with a confocal laser scanner, LuxScan™ 10 K (CapitalBio Corp.), and the resulting images analyzed with SpotData Pro 2.0 software (CapitalBio Corp.). Three biological replicates were performed. Differently expressed genes were identified using the *t*-test and multiple test corrections were performed using the False Discovery Rate (FDR) [42]. Genes with an FDR <0.01 and a fold change of double or more were considered to be different in gene expression.

For gene annotation, we used the updated TAIR (The Arabidopsis Information Resource) annotation for the Arabidopsis Genome Genechip array http://www.arabidopsis.org and the CapitalBio Corp MAS 2.0 system http://bioinfo.capitalbio.com/mas/. All data were submitted to the CapitalBio Corp MAS 2.0 system http://bioinfo.capitalbio.com/mas/. Genes were classified into functional categories using Gene Ontology information available from TAIR. The putative pathways were identified through the known pathways in the KEGG database provided by the CapitalBio Corp MAS 2.0 system http://bioinfo.capitalbio.com/mas/.

## Authors' contributions

JHY constructed the chimeric *orf220 *gene and transformed it to WT stem mustard. XYL carried out the localization of the chimeric *orf220 *gene. JHY and XDY analyzed the phenotype and pollen activity. The oligoarray experiment and analysis were proceeded at CapitalBio Corp. Beijing China. JHY wrote the paper. MFZ edited the paper. All the authors read and approved the final manuscript.

## Supplementary Material

Additional file 1 **Phenotype of flowering time and alterations on floral development in CMS stem mustard**. Click here for file

Additional file 2 **All down-regulated expressed genes detected in transgenic stem mustard**. Click here for file

Additional file 3 **All up-regulated expressed genes detected in transgenic stem mustard**. Click here for file
