# Supplementary material for: Mitochondrially-targeted expression of a cytoplasmic male sterility-associated orf220 gene causes male sterility in Brassica juncea
Source: BMC Plant Biol. 2010 Oct 26;10:231. doi: 10.1186/1471-2229-10-231 (PMC3017852; doi:10.1186/1471-2229-10-231)

**Additional files**

Additional file 1 - Phenotype of flowering time and alterations on floral development in CMS stem mustard. A: left, its maintainer fertile line; right, CMS. B: first flower, normal flower of its maintainer fertile line; the other flowers are of CMS. C: first pistil, normal pistil of its maintainer fertile line; the other pistils are of CMS.


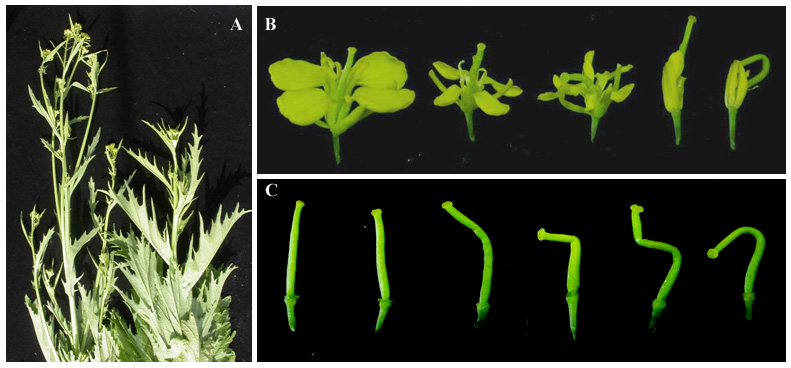

Supplement: Additional file 1 — Phenotype of flowering time and alterations on floral development in CMS stem mustard. [file 1471-2229-10-231-S1.DOC]
