# Supplementary material for: Mitochondrially-targeted expression of a cytoplasmic male sterility-associated orf220 gene causes male sterility in Brassica juncea
Source: BMC Plant Biol. 2010 Oct 26;10:231. doi: 10.1186/1471-2229-10-231 (PMC3017852; doi:10.1186/1471-2229-10-231)
Supplement: Additional file 2 — All down-regulated expressed genes detected in transgenic stem mustard. [file 1471-2229-10-231-S2.DOC]

**Additional files**

Additional file 2 - All down-regulated expressed genes detected in transgenic stem mustard detected by microarray

Table 1 All down-regulated expressed genes detected in transgenic stem mustard

| Gene ID | Gene Description | Fold |
| --- | --- | --- |
| At2g42940 | DNA-binding family protein | 2.5 |
| At2g07707 | hypothetical protein | 2.81 |
| At2g42840 | protodermal factor 1 (PDF1) | 4.6 |
| At1g75940 | glycosyl hydrolase family 1 protein / anther-specific protein ATA27 | 2.09 |
| At1g64600 | expressed protein | 2.43 |
| At1g01280 | cytochrome P450 family protein | 3.59 |
| At1g44970 | peroxidase, putative | 2.12 |
| At1g74640 | expressed protein / contain Pfam profile | 2.11 |
| At1g76470 | cinnamoyl-CoA reductase family | 3.41 |
| At3g07450 | protease inhibitor/seed storage/lipid transfer protein (LTP) family protein | 2.1 |
| At3g18430 | calcium-binding EF hand family protein | 2.03 |
| At3g13220 | ABC transporter family protein | 3.39 |
| At3g23770 | glycosyl hydrolase family 17 protein | 3.98 |
| At3g15270 | squamosa promoter-binding protein-like 5 (SPL5) | 2.96 |
| At3g11980 | male sterility protein 2 (MS2) | 3.48 |
| At3g06100 | major intrinsic family protein / MIP family protein | 3.51 |
| At1g54926 | hypothetical protein / hypothetical protein | 2.03 |
| At1g69500 | cytochrome P450 family protein | 3.89 |
| At1g20370 | tRNA pseudouridine synthase family protein | 2.08 |
| At4g12110 | sterol desaturase family protein | 4.56 |
| At4g22080 | pectate lyase family protein / pectate lyase family protein | 8.66 |
| At4g29660 | expressed protein | 2.79 |
| At4g30470 | cinnamoyl-CoA reductase-related | 2.42 |
| At4g34850 | chalcone and stilbene synthase family protein | 2.79 |
| At3g42960 | alcohol dehydrogenase (ATA1) | 3.62 |
| At5g22280 | expressed protein | 2.2 |
| cox2 | cytochrome c oxidase subunit 2 | 6.72 |
| cox1 | cytochrome c oxidase subunit 1 | 2.19 |
| orf111d | hypothetical protein | 2.53 |
| mitochondria.1 | ATP synthase subunit 9 | 2.06 |
