# Supplementary material for: Mitochondrially-targeted expression of a cytoplasmic male sterility-associated orf220 gene causes male sterility in Brassica juncea
Source: BMC Plant Biol. 2010 Oct 26;10:231. doi: 10.1186/1471-2229-10-231 (PMC3017852; doi:10.1186/1471-2229-10-231)
Supplement: Additional file 3 — All up-regulated expressed genes detected in transgenic stem mustard. [file 1471-2229-10-231-S3.DOC]

**Additional files**

Additional file 3 - All up-regulated expressed genes detected in transgenic stem mustard detected by microarray

Table 2 All up-regulated expressed genes detected in transgenic plant stem mustard

| Gene ID | Gene Description | Fold |
| --- | --- | --- |
| At2g42070 | MutT/nudix family protein | 2.28 |
| At2g02930 | glutathione S-transferase, putative /glutathione S-transferase, putative | 3.03 |
| At2g41250 | haloacid dehalogenase-like hydrolase family protein | 2.25 |
| At2g07560 | ATPase, plasma membrane-type, putative / proton pump, putative | 2.56 |
| At2g20900 | diacylglycerol kinase, putative | 2.14 |
| At1g78000 | sulfate transporter (Sultr1;2) | 2.11 |
| At1g50630 | expressed protein | 2.17 |
| At1g32780 | alcohol dehydrogenase, putative | 2 |
| At1g14420 | pectate lyase family protein | 2.12 |
| At1g52940 | calcineurin-like phosphoesterase family protein | 2.11 |
| At1g12240 | beta-fructosidase (BFRUCT4) / beta-fructofuranosidase / invertase, vacuolar | 2.04 |
| At1g02305 | cathepsin B-like cysteine protease, putative | 2.8 |
| At3g19930 | sugar transport protein (STP4) | 3.14 |
| At3g21710 | expressed protein | 4.16 |
| At3g14450 | RNA-binding protein, putative | 2.28 |
| At3g19090 | RNA-binding protein, putative | 2.21 |
| At1g69450 | early-responsive to dehydration protein-related / ERD protein-related | 2.19 |
| At4g25010 | nodulin MtN3 family protein | 2.27 |
| At4g25100 | superoxide dismutase (Fe), chloroplast (SODB) / iron superoxide dismutase (FSD1) | 2.57 |
| At4g28530 | no apical meristem (NAM) family protein | 2.2 |
| At4g29260 | acid phosphatase class B family protein | 2.14 |
| At4g39940 | adenylylsulfate kinase 2 (AKN2) | 2.06 |
| At3g48740 | nodulin MtN3 family protein | 2.06 |
| At3g57680 | peptidase S41 family protein | 2.02 |
| At3g61890 | homeobox-leucine zipper protein 12 (HB-12) / HD-ZIP transcription factor 12 | 2.13 |
| At5g12000 | protein kinase family protein | 2.48 |
| At5g16550 | expressed protein | 2.14 |
| At5g24580 | copper-binding family protein | 2.12 |
| At5g54080 | homogentisate 1,2-dioxygenase / homogentisicase/homogentisate oxygenase / homogentisic acid oxidase (HGO) | 2.09 |
| At5g25560 | zinc finger (C3HC4-type RING finger) family protein | 2.31 |
| At5g20710 | beta-galactosidase, putative / lactase, putative | 2.11 |
| At4g15530 | pyruvate phosphate dikinase family protein | 2.67 |
